# Unusual course of glyphosate-induced acute kidney injury: a case report of tubulointerstitial nephritis treated with steroids

**DOI:** 10.1007/s13730-024-00914-x

**Published:** 2024-07-29

**Authors:** Daichi Omote, Shin-ichi Makino, Issei Okunaga, Masayoshi Ishii, Narihito Tatsumoto, Masashi Aizawa, Katsuhiko Asanuma

**Affiliations:** Department of Nephrology, Graduate School of Medicine, Chiba University, 1-8-1 Inohana, Chuo-ku, Chiba-shi, Chiba, 260-8677 Japan

**Keywords:** Glyphosate, Tubulointerstitial nephritis, Acute kidney injury, Hemodialysis, Steroid therapy

## Abstract

Glyphosate is a widely used herbicide that is generally considered safe; however, acute kidney injury (AKI) caused by glyphosate ingestion can be severe and require hemodialysis. We present a unique case of a 68-year-old Japanese man who developed AKI after accidental ingestion of glyphosate and required hemodialysis. Based on the clinical presentation and findings, the patient was diagnosed with renal AKI with severe tubulointerstitial damage. However, the precise pathogenesis of the tubulointerstitial damage remained unclear. An elevated beta-2 microglobulin level discovered by the urinalysis during admission raised the suspicion of tubulointerstitial nephritis caused by glyphosate. Gallium scintigraphy revealed accumulation in both kidneys. A renal biopsy revealed acute tubulointerstitial nephritis rather than acute tubular necrosis, which is commonly observed with glyphosate-induced renal injury. After initiating steroid therapy, his kidney function gradually improved and he was weaned from hemodialysis. This report is the first to describe glyphosate-induced acute tubulointerstitial nephritis that was successfully treated with immunosuppressive therapy. Furthermore, this report highlights the importance of steroid therapy for cases of persistent kidney injury after the discontinuation of agents associated with acute tubulointerstitial nephritis.

## Introduction

Glyphosate-containing herbicides, which are available worldwide, are amino acid-based and non-selective. Although glyphosate is a relatively safe herbicide, large doses can cause abdominal pain, vomiting, mucosal irritation, corrosion, pulmonary edema, impaired consciousness, hypoxemia, and renal dysfunction, which can be fatal [1]. Hemodialysis is required for cases of severe renal dysfunction caused by glyphosate. During the general course of glyphosate-induced acute kidney injury (AKI), even with severe cases requiring hemodialysis, patients can be weaned from hemodialysis within a few days [2]. Here, we present the case of a patient who developed anuria and required hemodialysis after ingesting glyphosate. The patient was subsequently treated with steroids and weaned from dialysis 20 days after the treatment. This is the first report of successful steroid therapy for glyphosate-induced acute tubulointerstitial nephritis.

## Case report

A 68-year-old man presented with symptoms of anuria and dyspnea. He had a medical history of hypertension and hyperuricemia and was using cilnidipine, doxazosin mesylate, bisoprolol fumarate, and febuxostat. After retiring at 65 years of age, he consumed alcohol daily and accidentally ingested a small amount of glyphosate, approximately 30 ml, which is an herbicide, while intoxicated on day X-11, resulting in vomiting and diarrhea. As his symptoms persisted, he presented for medical attention on day X-3. Blood test results indicated decreased renal function; therefore, he was referred to the advanced emergency medical center. His serum urea nitrogen and creatinine levels were elevated (107 and 20 mg/dL, respectively). Despite receiving furosemide, the patient continued to experience anuria and required continuous hemodialysis. Because of persistent anuria, he was transferred to our hospital on day X for further examination and renal dysfunction treatment.

At the time of examination, generalized edema and respiratory failure with an oxygen saturation of 98% using an oxygen mask at 15 L/min were observed. The physical findings were unremarkable. There were no signs of ocular conjunctival pallor, yellowing, cough, sputum, or abdominal pain; however, edema of the trunk was observed. An arterial blood gas analysis revealed metabolic acidosis secondary to renal failure.

Further blood test results indicated a blood urea nitrogen level of 83 mg/dL, creatinine level of 15.6 mg/dL, and estimated glomerular filtration rate of 2.8 mL/min/m^2^, indicating markedly decreased renal function. He also had abnormal electrolytes, with a calcium level of 5.9 mg/dL and phosphorus level of 6.9 mg/dL, and a high inflammatory response, with a C-reactive protein level of 28 mg/dL and procalcitonin level of 3.68 ng/mL (Table 1).

**Table 1:** Laboratory findings at the time of admission

Urinalysis		Blood cell count		Serology	
pH	7.31	White blood cell, /μL	8500	C-reative protein, mg/dL	28
Protein	2 +	Hemoglobin, g/dL	11.5	Procalcitonin, ng/dL	3.68
Blood	2 +	Platelet, /μL	187,000	IgG, mg/dL	812
Sugar	2 +			IgA, mg/dL	170
Red blood cells, /HPF	50–99	Blood chemistry tests		IgM, mg/dL	59
Protein/creatine ratio, g/gCreatinine	6.3	Alanine aminotransferase, IU/L	19	C3, mg/dL	95
Sodium, mEq/L	102	Lactate dehydrogenase, IU/L	545	C4, mg/dL	38
Potassium, mEq/L	27	Alkaline phosphatase, IU/L	211	CH50, CH50/mL	68
Chloride, mEq/L	109	γ-Glutamyl transpeptidase, IU/L	65	Rheumatoid factor, IU/mL	Negative
NAG, U/L	40.6	Creatine kinase, IU/L	349	Anti-nuclear antibody	< x 40
β2-MG, μg/L	31,615	Total bilirubin, mg/dL	0.4	MPO-ANCA, U/mL	< 1.0
		Total protein, g/dL	4.8	PR3-ANCA, U/mL	< 1.0
		Albumin, g/dL	2.1	Anti GBM antibody, U/mL	< 7.0
		Uric acid, mg/dL	6.1	HBs antigen	Negative
		Blood urea nitorogen, mg/dL	83	Anti-HBs	Negative
		Creatinine, mg/dL	15.6	Anti-HBc	Negative
		eGFR-creatinine, mL/min/1.73 m^2^	2.8	Anti HCV	Negative
		Cystatin C, mg/L	4.54		
		eGFR-cystatin C, mL/min/1.73 m^2^	8.9	Blood gas(Artery,under administration of 15 L of oxygen)	
		Total cholesterol, mg/dL	138	pH	7.31
		Triglyceride, mg/dL	102	CO_2_, mmHg	29
		Sodium, mEq/L	140	O_2_, mmHg	66
		Potassium, mEq/L	3.8	HCO_3_^−^, mEq/L	14.6
		Chloride, mEq/L	107		
		Calcium, mg/dL	5.9		
		Inorganic-phosphonate, mg/dL	6.9		
		Glucose, mg/dL	112		
		Hemoglobin A1c, %	5.5		

*NAG* N-acetyl-β-D-glucosaminidase, *β2-MG* β-2 microglobulin, *eGFR* estimated glomerular filtration rate, *CH50* 50% hemolytic complement activity, *MPO-ANCA* myeloperoxidase antineutrophil cytoplasmic antibody, *PR3-ANCA* proteinase 3 antineutrophil cytoplasmic antibody, *GBM* glomerular basement membrane, *HBs* hepatitis B surface, *HBc* hepatitis B core, *HCV* hepatitis C virus, *pH* potential hydrogen, *PCO*_*2*_ partial pressure of carbon dioxide, *PO*_*2*_ partial pressure of oxygen, *HCO*_*3*_^*−*^ bicarbonate ion

The following results of the urinalysis indicated renal impairment: urine protein, 2 + ; urine occult blood, 2 + ; urine protein, 6.3 g/g creatinine; urinary red blood cells, 50–99 per high-power field. Tubulointerstitial damage was indicated by the following results: urinary sugar, 2 + ; urine N-acetyl-β-D-glucosaminidase, 40.6 U/L; and β-2 microglobulin, 31,615 μg/L (Table 1).

**Fig. 1:**
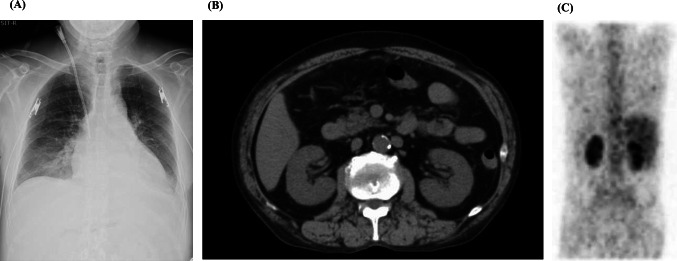
**A** Chest radiography shows decreased permeability of the lower lung fields in both lungs. **B** A CT scan shows that the kidney size is within the normal range. **C** Gallium scintigraphy shows abnormal accumulation of gallium in both kidneys, which is a finding of tubulointerstitial nephritis

Imaging studies included chest radiography, which showed a cardiothoracic ratio of 56%, blunting of the left costophrenic angle, and decreased permeability of the lower lung fields in both lungs (Fig. 1A). A simple computed tomography examination of the chest and abdomen revealed bilateral pleural effusions but no noticeable obstruction of the urinary tract, renal enlargement, atrophy, or neoplastic lesions. An ultrasound examination and a CT scan showed that the kidney size was approximately 10 cm, which was within the normal range (Fig. 1B).

Based on the clinical presentation and findings, the patient was diagnosed with AKI with severe tubulointerstitial damage. However, the precise pathogenesis of the tubulointerstitial damage remained unclear.

**Fig. 2:**
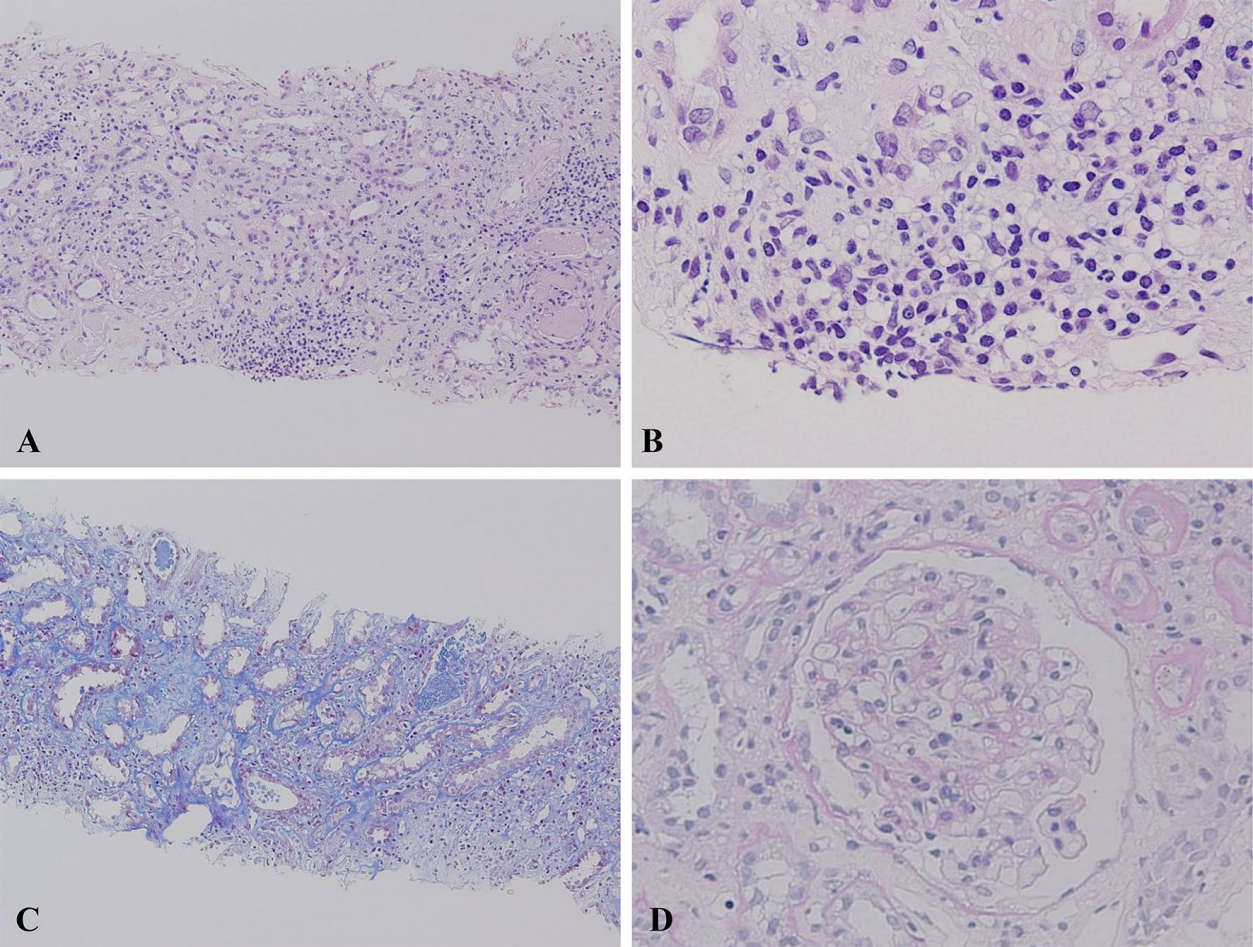
A renal biopsy was performed to diagnose tubulointerstitial nephritis. **A** Light microscopy (Hematoxylin–Eosin stain, 100 ×) shows tubular atrophy, columns, and lymphocytic infiltrate in the interstitium. **B** Light microscopy (Hematoxylin–Eosin stain, 400 ×) shows infiltration and aggregation of lymphocytes and plasma cells in the interstitium. **C** Light microscopy (Masson-Trichrome stain, 100 ×) shows mild stromal fibrosis and inflammatory cell infiltration into tubulointerstitium. **D** Light microscopy (Periodic and Schiff stain, 400 ×) shows no apparent damage to glomeruli

**Fig. 3:**
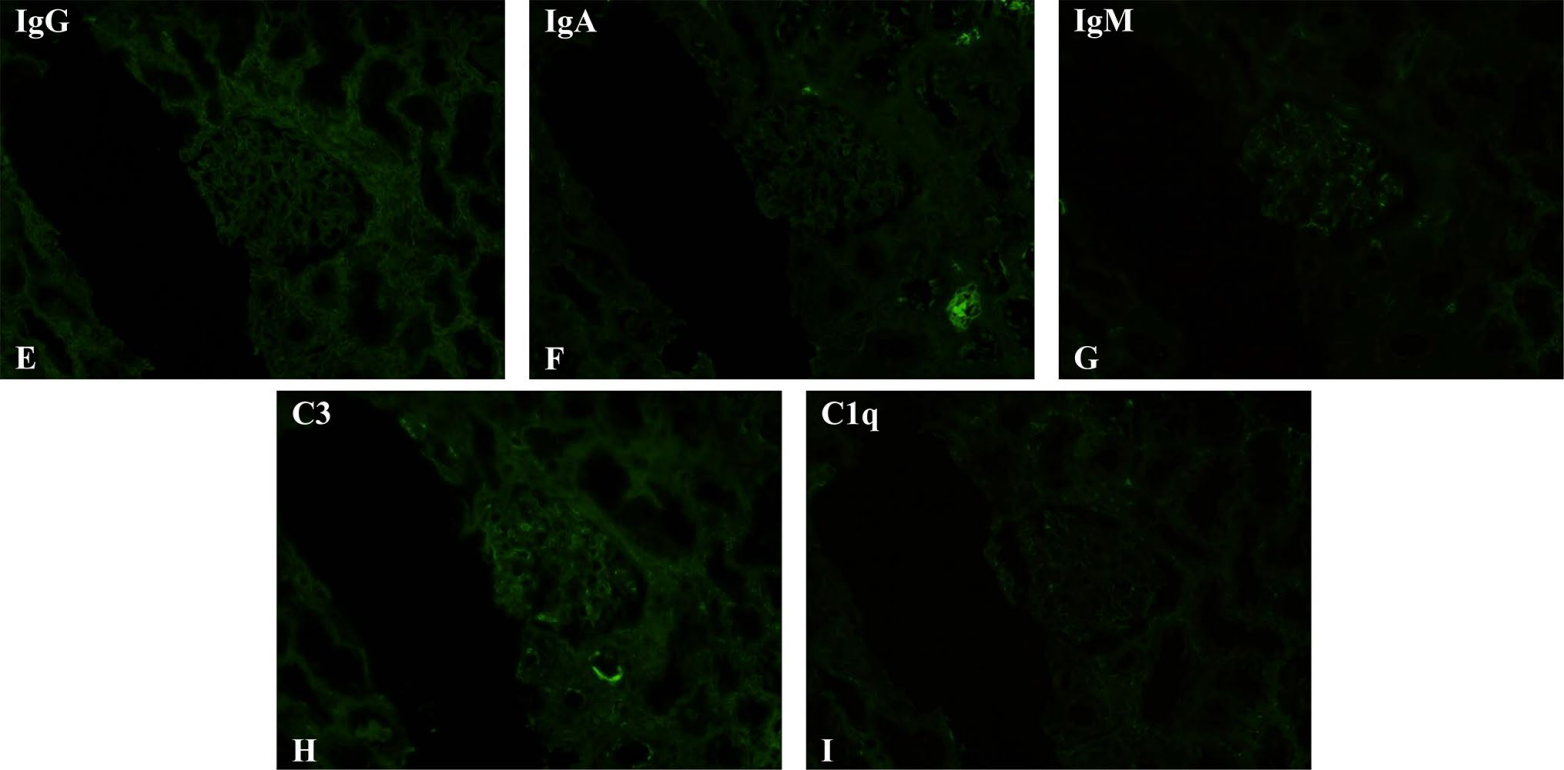
Immunofluorescence staining showed IgG, IgA, IgM, C3 and C1q were not deposited in the glomeruli. **E** Immunofluorescence staining of IgG(100 ×) **F** Immunofluorescence staining of IgA(100 ×) **G** Immunofluorescence staining of IgM(100 ×) **H** Immunofluorescence staining of C3(100 ×) **I** Immunofluorescence staining of C1q(100 ×)

**Fig. 4:**
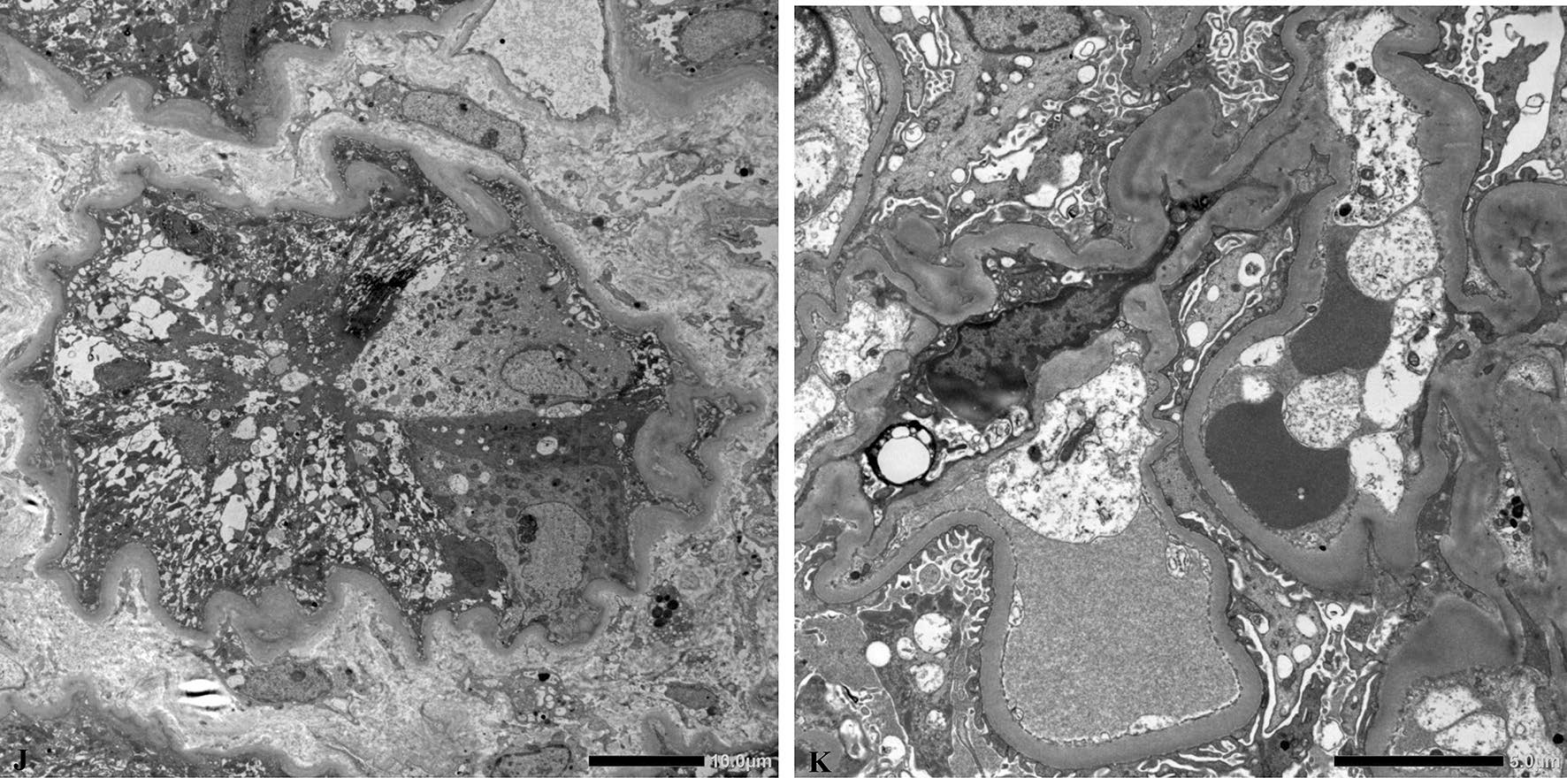
Electron microscopic findings. There were no dense deposits along the tubular basement membrane or in the glomerulus. **J** Election microscopy shows lymphocytes and monocytes are infiltrated within the proximal tubule. **K** Election microscopy shows no major changes in the glomerulus

**Fig. 5:**
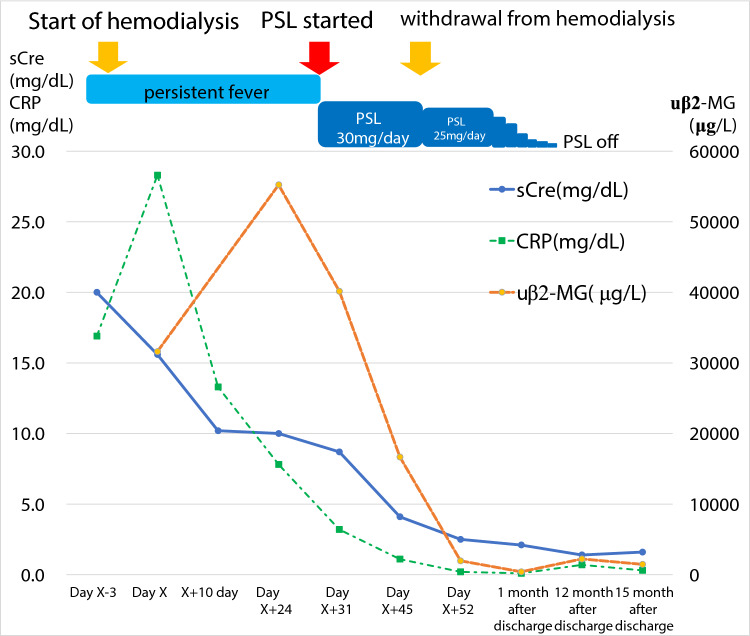
Clinical course of the present case. Serum creatinine (sCre), urine β-2 microglobulin (uβ2-MG), and CRP levels in blood tests before and after steroid treatment are shown. The patient was treated with steroids, and his renal function gradually improved. After 20 days of steroid therapy, he was weaned from dialysis and discharged

Upon admission, the patient required oxygen inhalation and a 15-L/min reservoir mask because of decreased oxygenation, fever, and pulmonary congestion, suggesting congestive heart failure and pneumonia. After transfer to our hospital, 4-h hemodialysis sessions were performed three times per week. With hemodialysis, respiratory failure improved to anoxic levels within 1 week. The fever persisted even after the respiratory condition improved. Transcatheter infection was suspected, cephazolin and vancomycin were administered, but the fever did not resolve, and various cultures did not detect any bacteria. Despite various tests, including contrast-enhanced computed tomography and echocardiography, the source of the fever remained unclear. An elevated β-2 microglobulin level discovered by the urinalysis during admission raised the suspicion of tubulointerstitial nephritis caused by glyphosate. Gallium scintigraphy revealed accumulation in both kidneys on day X + 24 (Fig. 1C). A renal biopsy performed on day X + 26 showed no significant glomerular damage, such as crescent formation, adhesion, segmental sclerosis, or expansion of the mesangial matrix; however, it revealed diffuse severe infiltration of lymphocytes in the tubulointerstitium, leading to a diagnosis of glyphosate-induced tubulointerstitial nephritis (Fig. 2A–D). Immunofluorescent staining showed that IgG, IgA, IgM, C3, and C1q were not deposited in the glomerulus (Fig. 3E–I). Electron microscopy showed that there were no dense deposits along the tubular basement membrane or in the glomerulus (Fig. 4J–K). After the renal biopsy, anuria persisted; therefore, prednisolone (PSL) 30 mg/day was started on day X + 28 for the treatment of acute tubulointerstitial nephritis. After the initiation of PSL therapy, the fever subsided and CRP decreased, and renal function gradually improved. At approximately day X + 44, the urine output increased to 1000 mL/day, thus allowing weaning from hemodialysis on day X + 47. His renal function continued to improve after the cessation of hemodialysis, and he was discharged on day X + 56. Thereafter, his renal function remained stable, with a serum creatinine level of approximately 1.5–2.0 mg/dL, and PSL treatment was gradually tapered and finished. Nephritis recurrence was not observed for more than 1 year (Fig. 5).

## Discussion

Glyphosate, which is an amino acid-based herbicide that is used worldwide, was registered as a pesticide in Japan in 1980. Ingestion of large amounts of glyphosate can induce a variety of symptoms, including gastrointestinal symptoms (vomiting and abdominal pain) caused by inflammation of the gastrointestinal mucosa, hypovolemic shock caused by increased vascular permeability, oliguria caused by renal failure, respiratory symptoms caused by associated pulmonary edema, and death. Glyphosate poisoning treatment is based on its symptoms. However, there is no established treatment for severe renal failure; therefore, hemodialysis should be initiated based on the criteria for AKI. Glyphosate concentrates in the kidneys and causes tubular necrosis, which results in kidney dysfunction [3]. In general, kidney damage is temporary, and weaning from dialysis, even for patients requiring hemodialysis, can be performed within a few days. Hemodialysis removes glyphosate from the blood and restores the urine volume [2, 4]. However, some case reports have described the death of the patient despite the initiation of hemodialysis [1, 5]. Unlike the findings reported in previous cases, persistent renal failure was observed in the present case because tubulointerstitial nephritis, rather than tubular necrosis, caused AKI. Generally, microscopic hematuria is not observed in tubulointerstitial nephritis, but microscopic hematuria in tubulointerstitial nephritis can be caused by damage to renal tubules and interstitium due to inflammatory reaction. In our case, microscopic hematuria was observed on urinalysis on admission, and serum creatinine and CRP were also elevated, suggesting that this was an effect of acute tubulointerstitial nephritis. Another case report of glyphosate-induced renal damage indicated that the kidney biopsy results revealed proximal tubular injury, and that the patient was weaned from hemodialysis without steroid treatment within 3 weeks [6]. In our case, the renal biopsy results revealed tubulointerstitial nephritis in addition to renal tubular necrosis. It is possible that the cause of TIN, rather than tubular necrosis, was simply a low dose of glyphosate. Interstitial nephritis persisted for more than 1 month after glyphosate administration, which could explain why the patient required steroid treatment. Although we could not rule out the possibility that the antibiotics administered after hospitalization may have triggered tubulointerstitial nephritis, this is the first case for which steroids were successfully used to treat tubulointerstitial nephritis after glyphosate-induced AKI.

Generally, with drug-induced tubulointerstitial nephritis, the drug itself is a small molecule that is not an antigen; however, it becomes an antigen when it binds to other substances (haptenization) and attacks activated immune cells, destroys tubular cells, or directly attacks tubular cells, such as the anticancer drug cisplatin, or when prostaglandin system inhibition causes tubular necrosis, as with nonsteroidal anti-inflammatory drugs [7]. Renal function declines relatively rapidly when the renal tubular interstitium is directly attacked. The diagnosis of tubulointerstitial nephritis is difficult; however, early detection of tubulointerstitial nephritis often improves renal outcomes. After diagnosis, 65% of patients recover their original renal function, and few experience progression to end-stage renal failure [8–10]. The early diagnosis of tubulointerstitial nephritis can be challenging, but it can restore renal function in 65% of patients, with only a few progressing to end-stage renal failure, as tubular damage is often reversible. In many drug-induced cases, recovery is achieved only by drug discontinuation. Steroids are indicated for drug-induced tubulointerstitial nephritis when there is no recovery of renal function after drug discontinuation, renal function declines rapidly, and renal biopsy tissue shows diffuse cellular infiltration and a small percentage of fibrosis [8–10]. Even in cases of persistent glyphosate-induced kidney injury, renal biopsy and the use of steroids should be aggressively considered. In our case, a renal biopsy was performed to diagnose tubulointerstitial nephritis. Light microscopy revealed tubular atrophy, cylinders, and lymphocyte and plasma cell infiltrates within the interstitium. In addition, mild interstitial fibrosis and inflammatory cell infiltration into the tubular interstitium, as well as partial tearing of the tubular basement membrane, were observed. Furthermore, there was no eosinophilic infiltrate, no flattening or vacuolar degeneration of the tubular epithelium, and no obvious damage to the glomerulus. Fluorescent antibody assay showed no significant deposition. Electron microscopy revealed no dense deposits along the tubular basement membrane or in the glomerulus, nor any mitochondrial damage. In a previous report of glyphosate-induced acute kidney injury, renal biopsy showed vacuolar degeneration of the proximal tubular epithelium and renal damage mainly due to tubular injury [6]. In this case, although the patient experienced glyphosate toxicity, the renal function improved after steroid therapy according to the criteria for steroid therapy for drug-induced tubulointerstitial nephritis. The reason for the significant response to steroids may be due to the fact that interstitial nephritis was the primary etiology. This case also showed that steroid therapy can improve the renal function of patients with tubulointerstitial nephritis who have not urinated for more than 1 month and require hemodialysis.

In conclusion, this case of glyphosate-induced tubulointerstitial nephritis required temporary hemodialysis because of severe renal impairment and anuria, and hemodialysis with steroid therapy was successfully weaned. This is the first case of glyphosate-induced acute tubulointerstitial nephritis successfully treated with immunosuppressive therapy. It demonstrates the importance of steroid therapy for cases of persistent renal injury after discontinuation of the agent that caused acute tubulointerstitial nephritis.
